# Intravenous fentanyl vs. topical lignocaine for ProSeal™ laryngeal mask airway insertion with propofol induction

**DOI:** 10.3389/fmed.2022.979275

**Published:** 2022-09-20

**Authors:** Nurzohara Aisha Noorazyze Rahmat Ameen Noorazyze, Nadia Md Nor, Jaafar Md Zain, Aliza Mohamad Yusof, Liu Chian Yong

**Affiliations:** ^1^Department of Anaesthesiology and Intensive Care, Hospital Raja Permaisuri Bainun, Ipoh, Malaysia; ^2^Department of Anaesthesiology and Intensive Care, Universiti Kebangsaan Malaysia Medical Centre, Kuala Lumpur, Malaysia

**Keywords:** lidocaine, laryngeal mask, propofol, anesthesia induction, fentanyl

## Abstract

Insertion of the laryngeal mask airway (LMA) without muscle relaxant requires adequate obtundation of airway reflexes, which may otherwise lead to incorrect or failed LMA placement. This study compared topical lignocaine spray vs. intravenous (IV) fentanyl, during propofol induction for insertion of the ProSeal™ LMA (PLMA). This was a prospective, randomized, double blind study, in ASA I or II patients, for elective or emergency surgery. Seventy patients (*n* = 70) who fulfilled the inclusion criteria were randomly assigned to receive IV fentanyl 2 mcg/kg or topical lignocaine spray 40 mg, prior to anesthesia induction with IV propofol (2–2.5 mg/kg). ProSeal™ LMA insertion condition was regarded optimal in the absence of adverse responses (gag, cough, laryngospasm and body movements), and successful LMA placement at the first attempt. Hemodynamic parameters were recorded and patients were assessed for sore throat and hoarseness post operatively. Seventy patients were analyzed. The number of patients with optimal PLMA insertion conditions were comparable between the groups (60% vs. 57%, *P* = 0.808). All hemodynamic parameters were comparable between groups with the exception of heart rate. Sympathetic obtundation of heart rate was greater with IV fentanyl than topical lignocaine (*P* < 0.05). The proportion of patients with postoperative sore throat significantly increased with the number of insertion attempts (*P* < 0.05). Topical lignocaine spray to the pharynx is as effective, and may be an alternative to IV fentanyl, during propofol induction for PLMA insertion. Success rate and optimal insertion condition at the first attempt, propofol requirement, blood pressure, adverse events and airway complications were comparable. Heart rate obtundation was less with topical lignocaine spray but remained within clinically acceptable values.

## Introduction

The laryngeal mask airway (LMA) is a supraglottic airway device introduced by Brain in 1983 (1). Its insertion does not require laryngoscopy, and supraglottic placement stimulates less airway reflex and sympathetic response than that associated with endotracheal intubation (2). However, adequate suppression of upper airway reflexes is required, as the LMA is usually inserted without muscle relaxant. Insufficient obtundation of airway reflexes may cause the patient to gag and cough, subsequently leading to incorrect LMA placement or insertion failure (3).

Studies have shown that propofol as an anesthetic induction agent provides superior LMA insertion conditions when compared to thiopentone, as it obtunds better the oropharyngeal and cough reflexes, and decreases sensitivity of the upper airway (4, 5). The recommended propofol dose for LMA insertion ranges from 2.5 to 3.5 mg/kg (6). Larger doses of propofol may cause cardio-respiratory depression, and using it as the sole anesthesia induction agent reduces the success rate of LMA insertion (5, 7).

Laryngeal mask airway insertion conditions are improved when propofol is used in combination with drugs such as midazolam, fentanyl, lignocaine and succinylcholine (5, 7). Opioids such as fentanyl decrease propofol requirement and improve LMA insertion conditions (7, 8). However, significant reductions in systolic and mean arterial blood pressures from baseline values, have been reported after fentanyl 2 μg/kg when compared to fentanyl 1 μg/kg, prior to propofol 2.5 mg/kg induction. Although, blood pressure reduction was not clinically relevant and did not require intervention, caution would have to be exercised in selected patients with poor cardiovascular status, where similar reductions in blood pressure could be clinically significant (9).

Topical and intravenous (IV) lignocaine have been used to obtund airway responses such as coughing and bucking during tracheal intubation (10, 11). Ahmed et al. showed that topical lignocaine spray 40 mg at the posterior pharyngeal wall 3 min before anesthesia induction with propofol 2 mg/kg, provided better LMA insertion conditions than propofol co-induction with IV lignocaine (12). Similarly, prior airway topicalization with lignocaine provided excellent LMA insertion conditions, with lower incidence of gag and cough, compared to IV midazolam (13).

Topical lignocaine provides surface anesthesia to the larynx and pharynx by cell membrane stabilization of the laryngeal and pharyngeal musculature, hence eliminating its sensitivity to airway stimulation during LMA insertion (14). Its anesthetic effect on the pharyngeal wall lasts 20–40 min (15), with lower peak plasma concentration than if it were administered parenterally, hence potentially reduces risk of systemic effects (16, 17).

Blood pressure and heart rate increase after LMA insertion, but were short-lived with values returning to baseline within a minute after airway stimulation (18, 19). Intravenous or topical lignocaine reduced the cardiovascular response to tracheal intubation and LMA insertion (10, 12). Baik et al. showed that hemodynamic stability was comparable between topical lignocaine 40 mg and IV lignocaine 1.5 mg/kg, and the former additionally improved LMA insertion conditions (20).

Airway instrumentation is a risk factor for postoperative sore throat, which is a common complaint post general anesthesia (21). Tanaka et al. showed that both topical and systemic lignocaine reduced the incidence of post-intubation sore throat (22).

There have been no studies comparing topical lignocaine vs. IV fentanyl, for LMA insertion. We compared topical lignocaine and IV fentanyl, prior to propofol induction, during insertion of the ProSeal™ LMA (PLMA). The PLMA is a second-generation LMA with a drainage tube which enables drainage of gastric secretions and content, and a rear cuff that allows higher seal pressure than a Classic LMA of equal intra-cuff pressure (23).

We hypothesized that topical lignocaine was as effective as IV fentanyl, before propofol induction, during PLMA insertion.

## Materials and methods

This prospective, randomized, double blinded study was carried out in the general operating theaters of Universiti Kebangsaan Malaysia Medical Centre (UKMMC). It was approved by the Dissertation Committee of the Anaesthesiology and Intensive Care Unit, UKMMC, and the Medical Research and Ethics Committee UKMMC (FF-2020-183; JEP-2019-828). We enrolled 70 patients of American Society of Anesthesiologist (ASA) I and II, aged between 18 and 65 years, who had surgery under general anesthesia with the PLMA. Patients with aspiration risk, allergy to the study drugs, body mass index (BMI) > 30 kg/m^2^, and cardiac arrhythmias were excluded.

Anesthesia medical officers were briefed on the study, and informed consent obtained from the patients. The patients were randomly allocated into two groups by computer generated randomization table, and fasted 6 h preoperatively. Group 1 patients received IV fentanyl and propofol, and Group 2 received topical lignocaine and propofol.

In the operation theater, standard monitoring which included the non-invasive blood pressure, electrocardiogram and pulse oximetry were applied, and baseline readings documented. The PLMA was lubricated with KY jelly on its dorsal cuff surface, and prepared for insertion with its curved metal introducer. The appropriate size PLMA was selected, based on the manufacturer's recommendation. ProSeal™ LMA insertion was performed by anesthetic medical officers with at least 2 years of experience in anesthesia.

The study drugs were prepared and administered by the investigator who was not blinded to the patient's group allocation. In the operation room, Group 1 patients received 2 ml normal saline (placebo), and Group 2 patients received 2 ml lignocaine 2% (40 mg), delivered *via* the MADgic™ laryngo-tracheal mucosal atomizer. This was done with the patient sitting, while their posterior pharyngeal wall was topicalized bilaterally before anesthesia induction.

Patients in both groups were then pre-oxygenated for 3 min, while allowing the onset of action of topical lignocaine. Induction of anesthesia proceeded in Group 1 with IV fentanyl 2 μg/kg and propofol 2–2.5 mg/kg, and in Group 2 with IV normal saline and propofol 2–2.5mg/kg. All patients were manually ventilated with 100% oxygen, and anesthesia was maintained with sevoflurane to achieve a minimum alveolar concentration (MAC) of 1–1.2. When pupils were central and constricted, and the jaw well relaxed, the PLMA was inserted by the anesthetic medical officer in charge. If during PLMA insertion the patient gagged or coughed, or if there was gross body movement, additional propofol bolus of 0.5 mg/kg was administered. Laryngospasm was managed with additional propofol 0.5 mg/kg bolus and increased sevoflurane concentration. Laryngospasm was defined as the presence of stridor, or other evidence of upper airway obstruction that subsides with deepening of anesthesia (24).

Successful placement of the PLMA was confirmed visually by adequate chest expansion bilaterally, and the capnograph on the monitor during spontaneous or assisted breathing. If the PLMA was malpositioned, it was removed and additional propofol 0.5 mg/kg bolus was administered before subsequent attempts. A maximum of three PLMA insertion attempts were allowed, after which further airway management was left to the discretion of the anesthesia medical officer in charge. These patients were considered as PLMA insertion failure, but were included in the study as PLMA insertion condition was only assessed during the first insertion attempt. Anesthesia was maintained with sevoflurane at 1–1.2 MAC, in 50% oxygen and air.

The anesthesia medical officer graded the first insertion attempt as optimal if there was absence of cough, gag, laryngospasm, or body movement, and when the PLMA was inserted successfully (24). ProSeal™ LMA insertion was graded as not optimal if one or more of the above adverse responses were present, or insertion was unsuccessful at the first attempt. The number of PLMA insertion attempts, PLMA insertion failure and total propofol required were documented.

Systolic blood pressure (SBP), diastolic blood pressure (DBP), mean arterial pressure (MAP), heart rate (HR) and oxygen saturation SpO_2_ were recorded by an assistant at pre-induction (baseline), post induction, immediately following ProSeal™ LMA insertion, and every minute thereafter for 5 min.

Postoperative airway complications of sore throat and voice hoarseness were assessed by the anesthesia medical officer after 30 min at the recovery area, and at 24 h postoperatively by the ward staff nurse, who were both blinded to the patient's group allocation. Sore throat was defined as “throat pain or discomfort” while hoarseness was defined as “a change in quality of voice” (25).

### Statistical analysis

Sample size was calculated using the computer program Sealed Envelope Ltd. 2012. Power calculator for binary outcome superiority trial was based on the Pocock formula 1983 (26). Gupta et al. compared IV fentanyl 1.5 μg/kg and propofol 2.5 mg/kg vs. ketamine/propofol and butorphanol/propofol combinations for anesthesia induction, and found excellent LMA insertion conditions in 43% of the patients in the fentanyl/propofol group (26). Ahmed S et al. found improved LMA insertion conditions in 83% of patients given topical lignocaine 40 mg and propofol 2 mg/kg, vs. those given IV lignocaine/propofol for anesthesia induction (11). Statistical analysis showed a significant difference between both results, *P* = 0.003. This study was powered at 95% and sample size calculated was 70 inclusive of a 20% dropout.

Statistical analysis was performed using the SPSS for Windows version 23.0 (IBM Corp, Armonk, NY, USA). The Chi-square test was used for categorical data analysis. Qualitative data was analyzed using the independent *t*-test for normally distributed data, and the Mann-Whitney U test for not normally distributed data. Results are presented as mean ± standard deviation, median (inter quartile range), or frequency (percentage) where appropriate. A *P* < 0.05 was considered statistically significant.

## Results

A total of 70 patients were recruited and there were no dropouts. Table 1 shows no difference in patient demographic between the groups.

**Table 1:** Patient demographics.

	**Group 1** **(*n* = 35)**	**Group 2** **(*n* = 35)**	***P*-value**
Age (year)	43.22 ± 16.41	45.94 ± 15.25	0.476
Gender (M/F)	13/22	11/24	0.615
Weight (kg)	66.14 ± 12.31	67.63 ± 13.78	0.636
Height (cm)	161.96 ± 9.28	161.00 ± 9.91	0.710
BMI (kg/m^2^)	25.08 ± 3.79	25.82 ± 3.72	0.412
ASA (1/2)	22/13	18/17	0.337

Values presented in mean ± standard deviation (SD), and number. P < 0.05 = significant.

The number of patients in which optimal insertion conditions were achieved at the first attempt, and the PLMA was successfully inserted at the first attempt, were comparable in both groups as shown in Table 2. Successful insertions at the first attempt were achieved in both groups despite not achieving optimal conditions for insertion. One patient in each group, with suboptimal PLMA insertion conditions, had failed PLMA insertion after three attempts.

**Table 2:** PLMA insertion attempts and insertion condition.

	**Group 1** **(*n* = 35)**	**Group 2** **(*n* = 35)**	***P*-value**
Successful insertion at 1st attempt	28 (80.0)	29 (82.8)	0.837
Optimal insertion conditions at 1st successful attempt	21 (60.0)	20 (57.1)	0.808
**Suboptimal insertions conditions**			
1st attempt successful insertion	7 (20.0)	9 (25.7)	0.789
2nd attempt successful insertion	5 (14.3)	3 (8.6)	0.789
3rd attempt successful insertion	1 (2.8)	2 (5.7)	0.789
Failed insertion	1 (2.8)	1 (2.8)	0.789

Values are presented in number (percentage). P < 0.05 = significant.

Table 3 shows no difference between the groups with regards to, adverse response during PLMA insertion at the first attempt, and post-operative airway complications. There was no incidence of laryngospasm in both groups.

**Table 3:** Adverse response during 1st attempt insertion and postoperative airway complications.

	**Group 1** **(*n* = 35)**	**Group 2** **(*n =* 35)**	***P*-value**
**Adverse responses at 1st attempt insertion:**			
Cough/gag	6 (17.1)	4 (11)	0.495
Body movement	12 (34.3)	13 (37)	0.803
Laryngospasm	0 (0.0)	0 (0)	
**Postoperative airway complications:**			
Sore throat in recovery	8 (22.8)	8 (22.8)	1.000
Sore throat at 24 h	1 (2.8)	0 (0.0)	0.314
Hoarseness in recovery	0 (0.0)	1 (2.8)	0.314
Hoarseness at 24 h	1 (2.8)	0 (0.0)	0.314

Values are presented in number (percentage). P < 0.05 = significant.

The proportion of patients with postoperative sore throat significantly increases with the number of ProSeal™ LMA attempts, as shown in Table 4.

**Table 4:** Post-operative complications and number of PLMA insertion attempts.

**Complications**	**1st attempt**	**2nd attempt**	**3rd attempt**	**Failed insertion**	***P*-value**
Sore throat in recovery	8 (14)	4 (50)	2 (67)	2 (100)	0.001*
Sore throat at 24 h	0 (0.0)	0 (0.0)	0 (0.0)	1 (50)	0.029*
Hoarseness in recovery	1 (2)	0 (0.0)	0 (0.0)	0 (0.0)	1.000
Hoarseness at 24 h	0 (0.0)	1 (12)	0 (0.0)	0 (0.0)	0.186

Values are presented in number (percentage). ^*^P < 0.05 = significant.

**Figure 1:**
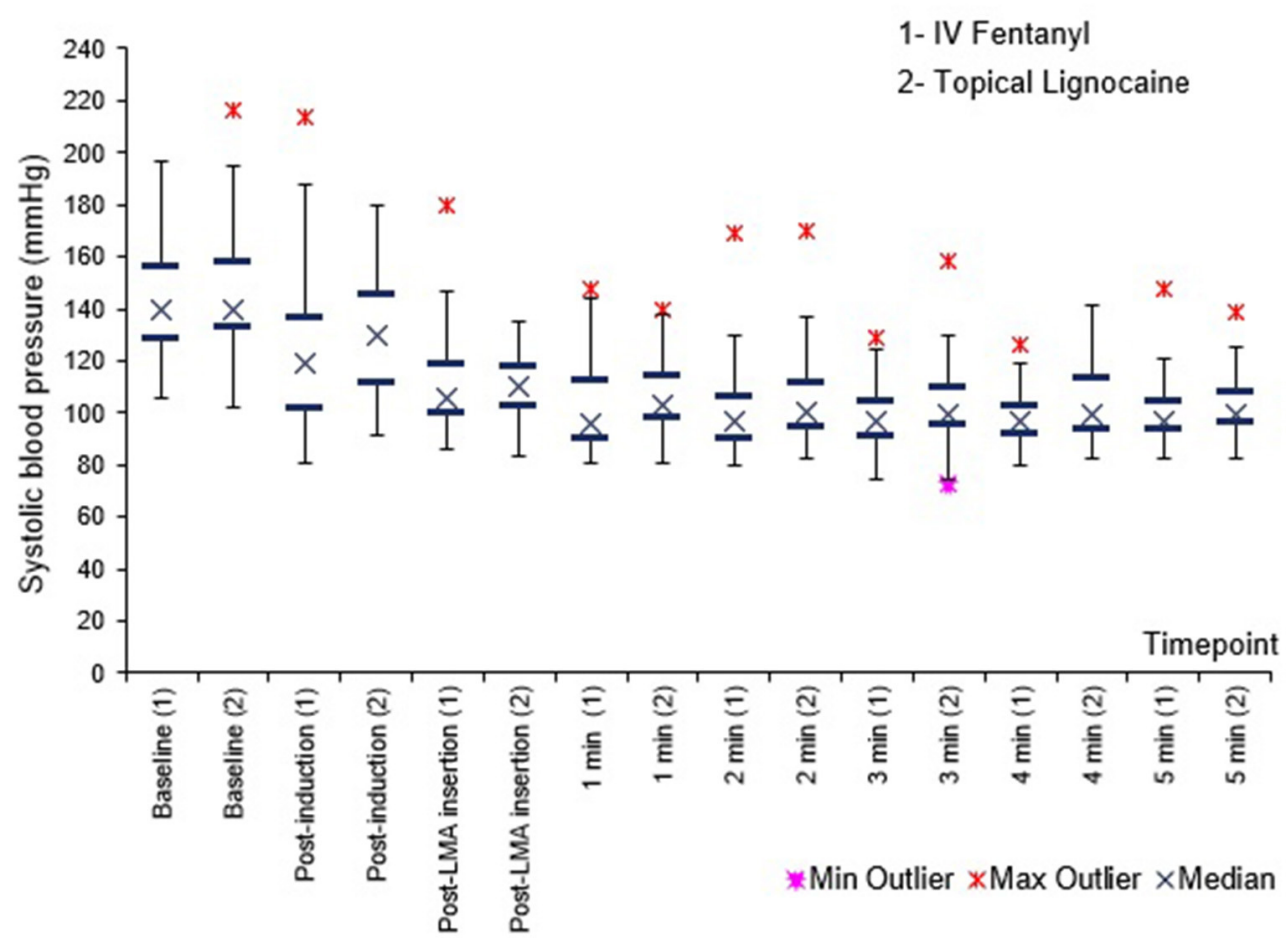
Systolic blood pressure (SBP) before, immediately after, and 1–5 min after PLMA insertion. Results presented as median, 25 and 75th percentile, minimum and maximum values.

**Figure 2:**
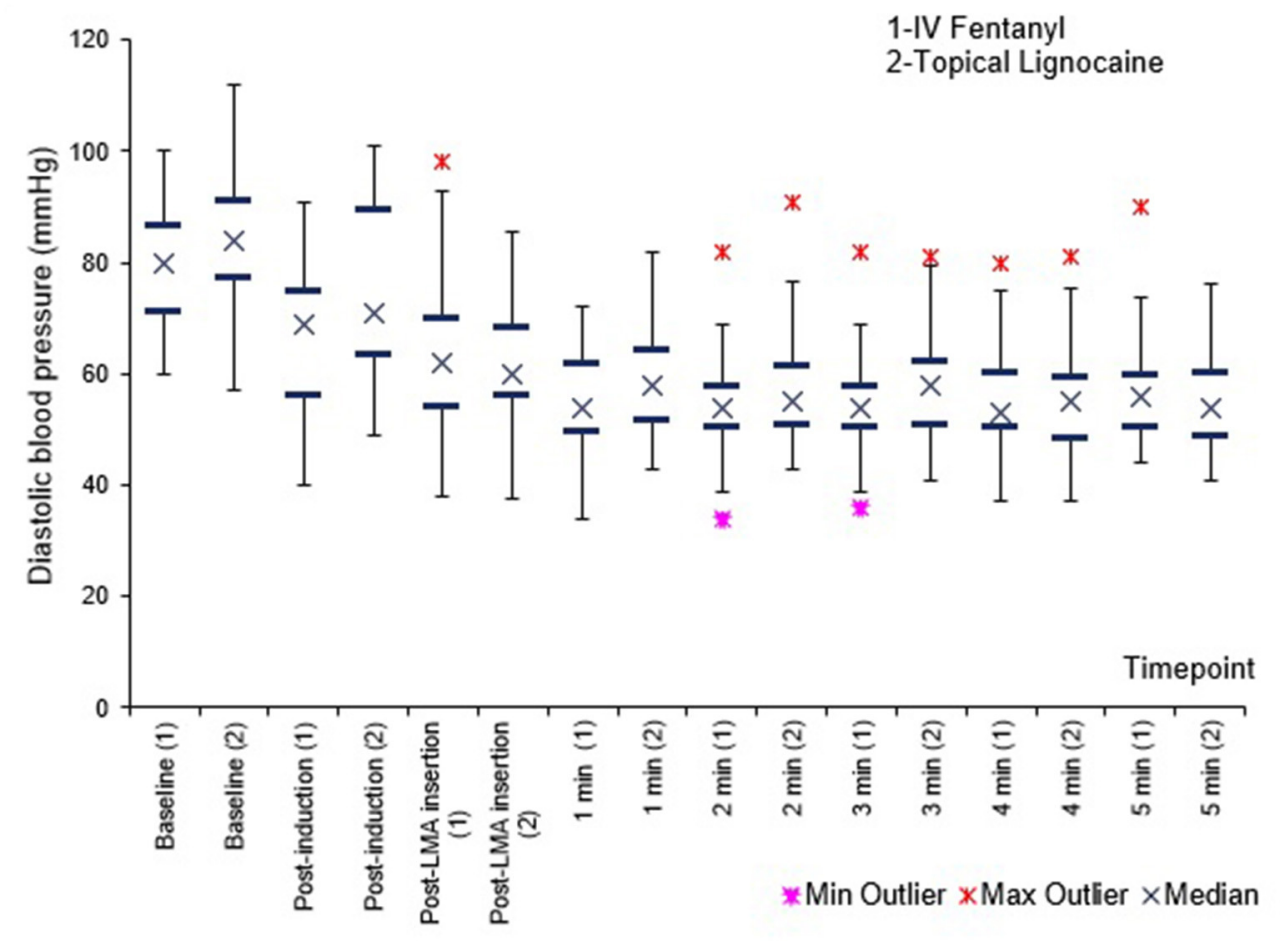
Diastolic blood pressure (DBP) before, immediately after, and 1–5 min after PLMA insertion. Results presented as median, 25 and 75th percentile, minimum and maximum values.

**Figure 3:**
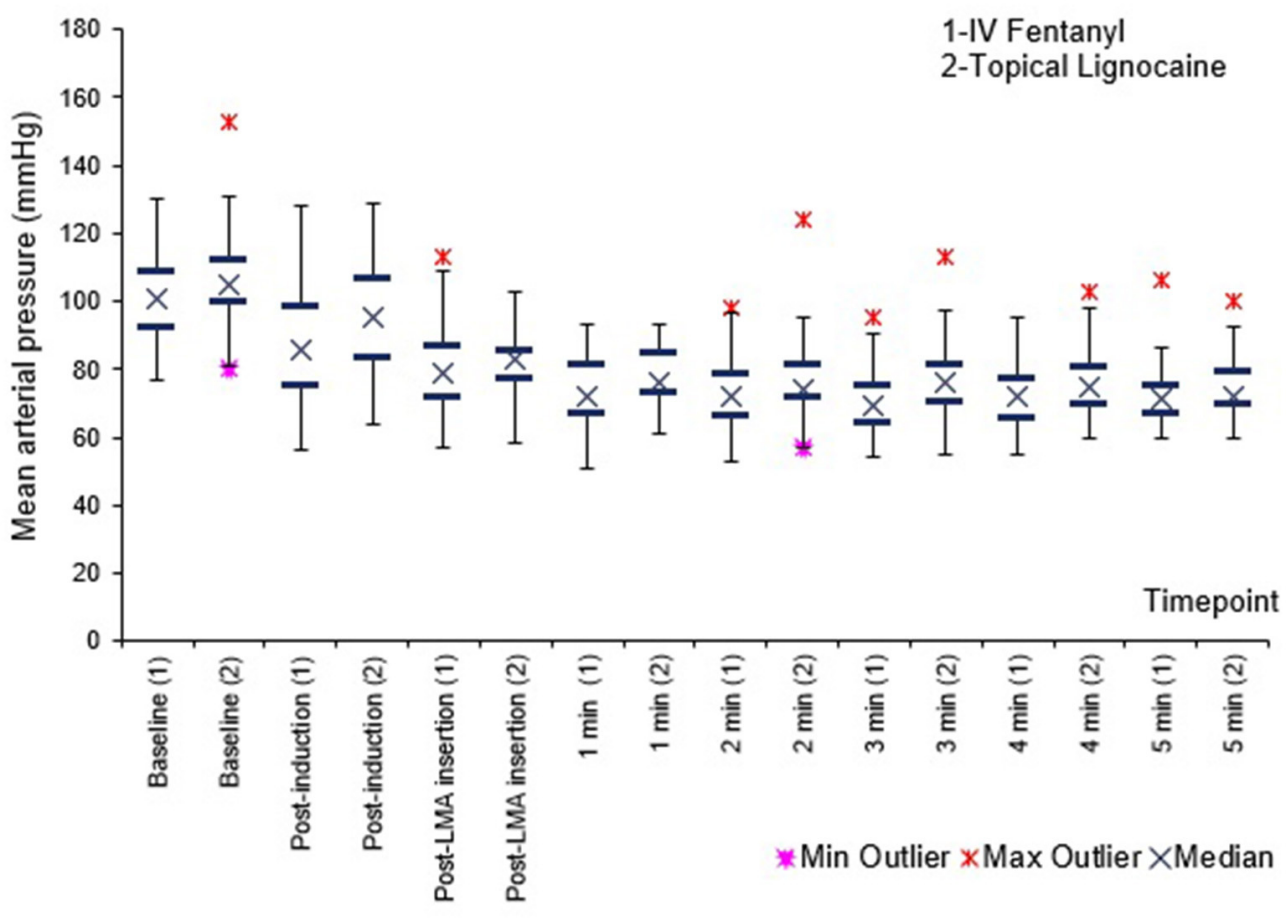
Mean arterial pressure (MAP) before, immediately after, and 1–5 min after PLMA insertion. Results presented as median, 25 and 75th percentile, minimum and maximum values.

Figures 1–3 shows no difference in SBP, DBP and MAP between the groups at all times.

**Figure 4:**
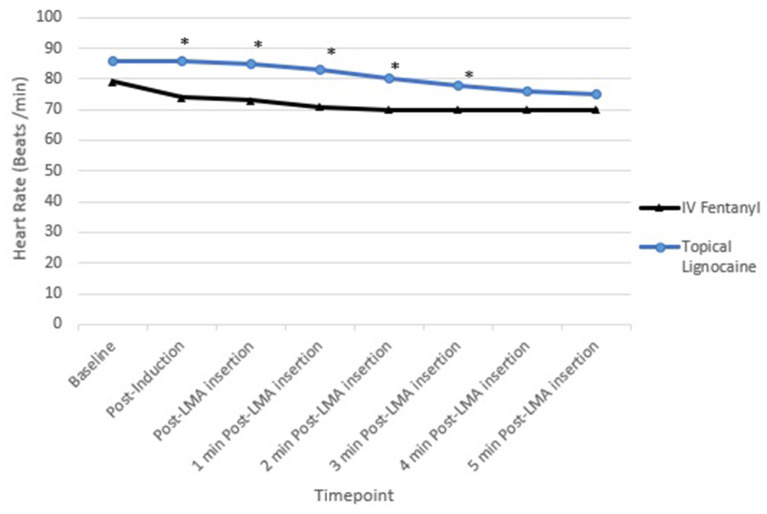
Mean heart rate (HR). *P < 0.05.

Figure 4 shows that sympathetic obtundation of heart rate was greater in the fentanyl group than the lignocaine group, from post induction until 3 min post PLMA insertion, *P* < 0.05.

Propofol requirement was comparable in both groups, at 2.14 (2.00–2.72) mg/kg and 2.50 (2.00–2.73) mg/kg, in the fentanyl and lignocaine groups, respectively, *P* = 0.379.

## Discussion

Laryngeal mask airways are usually inserted after anesthesia induction, without use of muscle relaxants. Various pharmacological agents have alternatively been used to facilitate LMA insertion (5, 7). Intravenous fentanyl is a frequently used opioid for co-induction during LMA insertion (28). However studies have also shown improved insertion conditions with prior topical pharyngeal lignocaine (12, 13, 24).

Optimal PLMA insertion conditions at the first attempt were achieved in about 60% of the patients in both groups. The numbers were comparable, suggesting similar efficacy of topical lignocaine and systemic fentanyl in achieving ideal PLMA placement conditions. Co-induction with fentanyl 2 μg/kg resulted in more of our patients (60%) achieving optimal insertion conditions, than that found in Gupta et al.'s study. The latter used a lower fentanyl dose of 1.5 μg/kg, and ideal insertion conditions were achieved in 43% of patients, despite their higher propofol dose of 2.5 mg/kg (27). When higher doses of fentanyl and propofol ranging 2–2.5 μg/kg and 2–2.5 mg/kg respectively were used, excellent LMA insertions conditions were attained in more than 80% patients (19, 29). However higher doses of fentanyl and propofol may compromise haemodynamic and respiration before the airway is secured. Rao and colleagues achieved optimal insertion conditions in more than 90% of their patients, with 100% success rate at first attempt LMA insertion. Viscous lignocaine gargle was given prior to co-induction with a lower dose of fentanyl 1 μg/kg and propofol 2 mg/kg (30).

We obtained a similar patient proportion (57%) with optimal insertion conditions, using topical lignocaine prior to propofol induction. This approximately mirrors findings by Changchien et al. and Seavell et al. who achieved optimal LMA insertion conditions with topical lignocaine 40 mg, in 66% and 73% of their patients respectively (24, 31). Changchien et al. additionally showed that subsequent anesthesia induction with propofol 2 mg/kg provided optimal LMA insertion conditions comparable to use of propofol 3 mg/kg alone, with the former having the added advantage of reduced incidence of apnea and cardiovascular instability (24). Shazed M. et al. achieved optimal insertion conditions in over 98% of their patients who were administered 200 mg topical lignocaine, which is higher than that utilized in most studies (32).

Successful PLMA insertion at the first attempt was achieved in more than 80% of the patients, and this was comparable between the groups. This was in concordance with a prior study which achieved first attempt success in more than 90% of patients in both fentanyl and topical lignocaine groups (8, 24). Optimal insertion conditions were not achieved in all successful insertions at the first attempt. This was evident in both groups, where the percentage of successful first attempt insertions exceeded the percentage of patients in which optimal conditions were achieved during the first attempt. This may imply that the presence of adverse events, potentially leading to poor PLMA insertion conditions, may not necessarily hamper successful placement. The risk of failed LMA insertion has also been shown to increase with advanced age, high body weight, BMI <20 kg/m^2^ and insertions without lignocaine gel (33).

Insufficient obtundation of airway reflexes may trigger gag and cough reflexes which could lead to incorrect LMA placement or insertion failure (3). We found comparable incidence of cough and gag with topical lignocaine (11%) and fentanyl (16%), which were in concordance with that found by Changchien et al. and Dhamotharan et al. with cough and gag reflexes in 10 and 16% of their patients administered topical lignocaine and IV fentanyl respectively (24, 29). In the study by Shazed M, the combination of a higher dose of 200 mg topical lignocaine, followed by the synergistic effects of IV nalbuphine co-induction with propofol 2 mg/kg provided a deeper plane of anesthesia and attenuation of upper airway reflexes, with reduced gag incidence of 3.5% (32). None of our patients developed laryngospasm. Suppression of laryngospasm by prior administration of fentanyl or topical lignocaine was appreciated in studies by Cheam et al. and Changchien et al., respectively (8, 24). The incidence of body movements was also comparable between the groups, and similarly so in prior studies (8).

Traumatic insertion of the LMA may cause post-operative sore throat and is preventable with smooth LMA insertion (8). About a fifth of our patients in both groups developed sore throat in recovery, which was short-lived. This was in concordance with a study by Kuppusamy et al., where 25% of their patients had sore throat with PLMA insertion (34). Only one of our patients in the fentanyl group had symptoms persisting at the 24th hour postoperatively. Our study showed that the proportion of patients with postoperative sore throat significantly increased with the number of PLMA attempts (*P* < 0.05), and this was consistent with a prior study by Grady et al. (25). One patient in each group experienced hoarseness, at recovery and at 24 h respectively.

Haemodynamically, SBP, DBP and MAP were comparable between the groups at all times. Heart rate was lower in the fentanyl group than the lignocaine group from post induction up to 3 min post PLMA insertion, *P* < 0.05. Sympathetic obtundation of HR was greater with fentanyl than lignocaine. An earlier study showed similar finding with incidence of bradycardia (29).

Propofol requirement was not significantly different between the groups. Fentanyl 2 μg/kg has been shown to reduce propofol requirement by 60% during LMA insertion (7). Median propofol dose in our patient group given topical lignocaine was 2.50 (2.00–2.73) mg/kg. Topical lignocaine produced excellent LMA insertion conditions during anesthesia induction with propofol 2 mg/kg (12, 24). Higher propofol doses of 2.5–3.5 mg/kg was required if used as a sole anesthesia induction agent (6).

Limitations of this study include possible differences in the individual skills of the medical officer in PLMA insertion, thus confounding results of first attempt success rates and incidence of adverse effects. A single operator performing PLMA insertions may have reduced this bias. Other factors not considered in this study were reduced mouth opening (inter-incisor distance <3 cm), higher Mallampati grade (III, IV), reduced neck mobility, age of >61 years, and BMI of <20 kg/m^2^, all of which could have also confound the successful placement of the PLMA (33, 35).

Topical lignocaine spray to the pharynx is as effective, and may be an alternative to IV fentanyl, during propofol anesthesia induction for PLMA insertion. The success rate at first attempt, optimal insertion conditions, propofol requirement, blood pressure, adverse events and airway complications were comparable. Heart rate obtundation was less with topical lignocaine spray, but remained within clinically acceptable values.

## Data availability statement

The raw data supporting the conclusions of this article will be made available by the authors, without undue reservation.

## Ethics statement

The studies involving human participants were reviewed and approved by Ethics Research Secretariat, Universiti Kebangsaan Malaysia Medical Centre, Malaysia. The patients provided their written informed consent to participatein this study.

## Author contributions

NN: article concept, design, intellectual content, literature search, data and statistical analysis, manuscript preparation, and editing and review. NR: article concept, design, intellectual content, literature search, data acquisition, data and statistical analysis, manuscript preparation, and editing and review. JZ, AM, and LY: intellectual content, literature search, data and statistical analysis, manuscript preparation, and editing and review. All authors contributed to the article and approved the submitted version.

## Funding

Funding of this research was obtained from Universiti Kebangsaan Malaysia fundamental grant with Project Code No: FF-2020-183.

## Conflict of interest

The authors declare that the research was conducted in the absence of any commercial or financial relationships that could be construed as a potential conflict of interest.

## Publisher's note

All claims expressed in this article are solely those of the authors and do not necessarily represent those of their affiliated organizations, or those of the publisher, the editors and the reviewers. Any product that may be evaluated in this article, or claim that may be made by its manufacturer, is not guaranteed or endorsed by the publisher.
